# Development of the MTB/IC LAMP-MS Assay for Rapid Detection of *Mycobacterium tuberculosis*

**DOI:** 10.3390/diagnostics15080996

**Published:** 2025-04-14

**Authors:** Woong-Sik Jang, Seoyeon Park, Jun-min Lee, Eunji Lee, Chae-Seung Lim

**Affiliations:** 1 Emergency Medicine, College of Medicine, Korea University Guro Hospital, 148, Gurodong-ro, Guro-gu, Seoul 08308, Republic of Korea; plasmid18@korea.ac.kr; 2 Department of Laboratory Medicine, College of Medicine, Korea University Guro Hospital, 148, Gurodong-ro, Guro-gu, Seoul 08308, Republic of Korea; 08tjdus@naver.com; 3 BK21 Graduate Program, Department of Biomedical Sciences, College of Medicine, Korea University, 145 Anam-ro, Seongbuk-gu, Seoul 02841, Republic of Korea; dlwnsals15@korea.ac.kr (J.-m.L.); lucy5303@korea.ac.kr (E.L.)

**Keywords:** loop-mediated isothermal amplification, *Mycobacterium tuberculosis*, point-of-care testing, rapid TB detection

## Abstract

**Background:** Tuberculosis (TB) remains a significant global health challenge, necessitating rapid and reliable diagnostic solutions. Current methods such as microscopy, culture, and PCR have limitations in resource-limited settings due to high costs and infrastructure requirements. **Methods:** In this study, we developed the MTB/IC LAMP-MS assay, a novel molecular diagnostic platform that integrates loop-mediated isothermal amplification (LAMP) technology with microscope (MS) detection. The assay enables TB diagnosis within 40 min, from nucleic acid extraction to result, requiring minimal equipment and simple operation for rapid and accurate detection. **Results:** Compared to the commercial AdvanSure TB/NTM real-time PCR kit, the MTB/IC LAMP-MS assay had a lower analytical sensitivity, with a limit of detection of 10^3^ CFU/mL versus 10^2^ CFU/mL for the commercial PCR kit, but showed a sensitivity of 93.3% and specificity of 100% in clinical tests. Additionally, the assay demonstrated minimal cross-reactivity with other respiratory pathogens, underscoring its robust specificity. In addition, the preloaded microchips demonstrated stable performance for up to 12 weeks at room temperature, supporting their field applicability. **Conclusions:** With its lower cost, simplified operation, and minimal infrastructure requirements, the MTB/IC LAMP-MS assay provides a practical and efficient solution for TB diagnosis in resource-limited settings.

## 1. Introduction

Tuberculosis (TB) remains a major global health challenge, contributing to substantial morbidity and mortality worldwide [1]. According to the World Health Organization (WHO), approximately 10.6 million people developed TB in 2022, with 1.6 million deaths attributed to the disease [2]. The causative pathogen, *Mycobacterium tuberculosis* (MTB), primarily infects the lungs, but without early diagnosis and timely treatment, it can lead to severe disease progression and increased transmission [3]. The rising prevalence of multidrug-resistant TB (MDR-TB) and extensively drug-resistant TB (XDR-TB) has further complicated TB management, underscoring the urgent need for rapid, accurate, and accessible diagnostic methods [4,5,6].

Conventional TB diagnostic methods include acid-fast bacilli (AFB) smear microscopy, mycobacterial culture, and nucleic acid amplification tests (NAATs). Smear microscopy is widely used due to its simplicity, rapid turnaround time, and low cost; however, it has low sensitivity (40–60%), making it unreliable, particularly in immunocompromised patients, where sensitivity drops to 20–40% [7,8,9]. Culture remains the gold standard for TB diagnosis, but the slow growth of MTB results in delayed diagnoses (several weeks), leading to treatment delays and continued disease transmission [10,11]. In contrast, NAAT-based diagnostic methods, such as the Xpert MTB/RIF assay (Cepheid, USA), offer high sensitivity (>95%) and specificity (>98%), enabling rapid MTB detection and simultaneous rifampin resistance profiling [8,12,13]. However, these molecular assays require advanced laboratory infrastructure and high costs, which significantly limit their accessibility in resource-constrained settings [14,15]. To overcome these limitations, isothermal amplification technologies, particularly loop-mediated isothermal amplification (LAMP), have emerged as promising alternatives for TB diagnosis. LAMP enables rapid and highly sensitive DNA amplification at a constant temperature (60–65 °C), eliminating the need for thermal cycling equipment [16,17,18]. Due to these advantages, the WHO has endorsed the TB-LAMP assay (Eiken Chemical, Tokyo, Japan) as an alternative to smear microscopy, demonstrating high sensitivity even in HIV co-infected patients [19]. However, conventional LAMP-based diagnostic methods often require additional detection systems, such as fluorescence or turbidity measurements, which can increase complexity and costs. While colorimetric detection allows for visual interpretation, additional devices may be needed when distinguishing positive and negative results is challenging [20].

To address these challenges, we developed the MTB/IC LAMP-microscopy (MS) assay, a novel molecular diagnostic platform integrating LAMP technology with microscopy-based detection. This assay is a slightly modified version of our previously developed LAMP-microscanner (MS) technology, originally designed for SARS-CoV-2 detection, in which an automated microscanner was used to visualize magnesium pyrophosphate byproducts and confirm amplification [21]. This method enables the rapid and intuitive interpretation of results without the need for fluorescence detection or complex analytical instruments, making it a cost-effective and field-deployable alternative to conventional LAMP-based detection methods. Additionally, the incorporation of a microfluidic reaction chip enhances stability, ensuring the assay’s suitability for resource-limited settings. In this study, we evaluated the analytical and clinical performance of the MTB/IC LAMP-MS assay and compared it with the commercial AdvanSure TB/NTM real-time PCR kit (LG Lifescience, Seoul, Republic of Korea).

## 2. Materials and Methods

### 2.1. Clinical Samples

*Mycobacterium tuberculosis* (KCTC 39720) was obtained from the Korean Collection for Type Cultures (KCTC) for the limit of detection (LOD) analysis. The clinical sensitivity assessment was conducted using 190 residual clinical samples collected during routine diagnostic testing at Korea University Guro Hospital between July 2021 and November 2023. These samples comprised 77 DNA and 13 sputum specimens from tuberculosis-confirmed patients and 100 DNA specimens from non-infected control individuals. All samples were stored at −70 °C until further analysis. Tuberculosis infection was confirmed using the AdvanSure™ TB/NTM RT-PCR kit (LG Life Sciences, Seoul, Republic of Korea). For cross-reactivity evaluation, 14 residual nasopharyngeal (NP) swab samples collected from patients diagnosed with respiratory viral infections at Korea University Guro Hospital were used. These infections included influenza virus subtypes (A H1, A H1N1, A H3, B), coronaviruses (HKU1, NL63, 229E), adenovirus (AdV), respiratory syncytial virus A (RSV A), parainfluenza virus subtypes (PIV 1–4), and human rhinovirus (HRV), confirmed using the Anyplex II RV16 Detection Kit (Seegene Inc., Seoul, Republic of Korea). In addition, *Legionella pneumophila*, *Streptococcus pneumoniae*, *Klebsiella pneumoniae*, *Pseudomonas aeruginosa*, *Streptococcus pyogenes*, *Mycobacterium intracellulare*, *M. avium*, *M. fortuitum*, and *M. kansasii* were obtained from the Department of Laboratory Medicine at Korea University Guro Hospital. Furthermore, *M. abscessus* (KCTC19621), *M. chelonae* (KCTC29796), and *M. massiliense* (KCTC19086) were obtained from the Korean Collection for Type Cultures (KCTC) for cross-reactivity testing. This study was conducted in accordance with the guidelines of the Declaration of Helsinki and approved by the Institutional Review Board of Korea University Guro Hospital (Approval No.: 2021GR0550).

### 2.2. DNA Extraction

Genomic DNA from clinical samples was extracted using two distinct methods: the QIAamp DNA Mini Kit (QIAGEN, Hilden, Germany) and a Chelex-100/boiling method. While the Qiagen kit offers high efficiency and reliability, the Chelex-100 method provides a cost-effective alternative suitable for resource-limited settings. For the Qiagen-based extraction, 200 µL of each clinical sample was processed following the manufacturer’s protocol, including lysis, washing, and elution steps using the QIAamp Mini spin column. The Chelex-100 method involved adding 10 µL of each clinical sample into custom-made tubes containing 190 µL of 10% Chelex-100 resin solution (10 mM Tris-HCl, 1 mM EDTA, pH 8.0) [22]. The tubes were securely capped and incubated at 100 °C for 5 min to lyse cells. Following incubation, samples were compressed through a 0.45 µm filter built into the tube cap, separating the Chelex-100 resin from the nucleic acid solution. Approximately 100 µL of the resulting DNA-containing supernatant was collected for subsequent analysis.

### 2.3. The MTB/IC LAMP-MS Assay

The primer sets for TB and the internal control (IC) used in the LAMP-MS assay were previously developed and reported by our research group [23] (Table 1). The TB primer set targets the IS6110 gene specific to *M. tuberculosis*, while the IC primer set targets the conserved region of the human ACTB (actin beta) gene. All primers were synthesized by Macrogen Inc. (Seoul, Republic of Korea). Each primer mix contained two outer primers (F3 and B3) at 4 µM, two inner primers (FIP and BIP) at 32 µM, and two loop primers (LF and LB) at 10 µM. A volume of 0.5 µL of each primer set (TB and IC) was individually loaded into separate channels of a microchip (Biozentech, Seoul, Republic of Korea). The microchip was dried in a sterilized oven at 58 °C for 30 min and stored in a sealed container at room temperature until use. The reaction mixture for the MTB/IC LAMP-MS assay consisted of 12.5 µL of Mmiso^®^ DNA amplification kit Master Mix (Mmonitor, Daegu, Republic of Korea), 2 µL of Mmiso^®^ DNA amplification kit Enzyme (Mmonitor, Daegu, Republic of Korea), and 5 µL of DNA template, resulting in a final reaction volume of 25 µL. The prepared reaction mixture was individually loaded into each microchip channel at 10 µL per channel, followed by amplification at 62 °C for 30 min using a heating block (Beijing HiYi Technology, Beijing, China). The inlet and outlet of the microchip were sealed with sterile adhesive to prevent evaporation during amplification. After the LAMP reaction, the microchip was analyzed using an optical RV400TMCL microscope (OMAX OMB microscope, Bucheon, Republic of Korea) at 20× magnification to confirm the presence of amplification products. DNA amplification was detected by visualizing magnesium pyrophosphate precipitate, a byproduct of the LAMP reaction, facilitated by the embedded grid within the microchip.

### 2.4. The AdvanSure™ TB/NTM Real-Time PCR

To evaluate the performance of the MTB/IC LAMP-MS assay, a comparative analysis was conducted using the AdvanSure™ TB/NTM real-time PCR. The AdvanSure^TM^ TB/NTM real-time PCR was performed on the SLAN real-time PCR system from LG Life Sciences in Seoul, Republic of Korea, following the manufacturer’s instructions. For the PCR reaction, 5 μL of the TB/NTM primer–probe mixture and 5 μL of DNA sample were added to each PCR tube containing 10 μL of a 2×PCR mixture. The PCR cycling conditions were as follows: the initial inactivation step was performed at 50 °C for 2 min, followed by denaturation at 95 °C for 10 min. The PCR then underwent 35 cycles of denaturation at 95 °C for 10 s and annealing with fluorescence detection at 62 °C for 40 s.

### 2.5. Limit of Detection Tests of the MTB/IC LAMP-MS Assay

To determine the limit of detection of the MTB/IC LAMP-MS assay, *Mycobacterium tuberculosis* strain KCTC 39720, with an initial bacterial concentration of 1 × 10^8^ CFU/mL, was diluted in non-infected nasopharyngeal clinical samples to achieve a final concentration of 1 × 10^6^ CFU/mL. A ten-fold serial dilution was then performed using non-infected nasopharyngeal clinical samples to prepare five different bacterial concentrations, ranging from a high concentration of 1 × 10^5^ CFU/mL to a low concentration of 1 × 10^1^ CFU/mL. The limit of detection (LOD) of the TB/IC LAMP-MS assay was evaluated following nucleic acid extraction. Each test was performed in 20 replicates. The detection limit was defined as the lowest bacterial concentration at which all 20 replicates produced positive results.

### 2.6. Stability Tests of the MTB/IC LAMP-MS Assay

To evaluate the stability of the MTB/IC LAMP-MS assay, *Mycobacterium tuberculosis*-positive and *Mycobacterium tuberculosis*-negative samples were used for testing. Microchips preloaded with primers in each channel were dried in an oven and then stored at room temperature for up to 12 weeks to assess their long-term stability. A total of four samples were used in the tests, which included two *Mycobacterium tuberculosis*-positive samples and two *Mycobacterium tuberculosis*-negative samples. The same samples were tested at seven different time points, including days 1, 3, 7, 14, 28, 56, and 84. The results of each test were recorded as either positive or negative.

## 3. Results

### 3.1. Development of the MTB/IC LAMP-MS Assay

**Figure 1:**
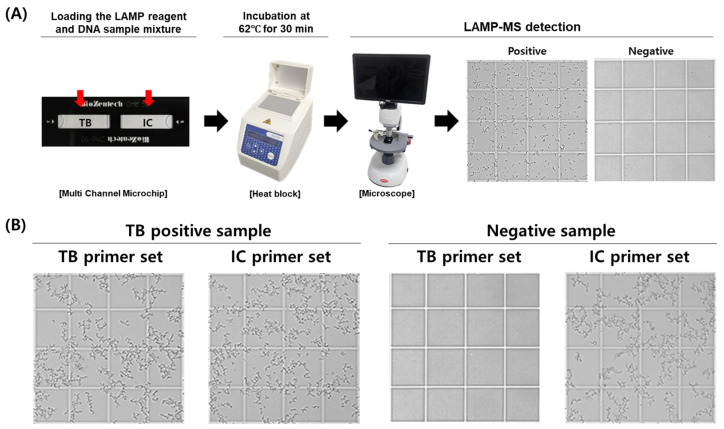
Workflow and representative results of the MTB/IC LAMP-MS assay. (**A**) Schematic workflow of the MTB/IC LAMP-MS assay. A total of 20 μL of LAMP reaction mixture containing the DNA sample is prepared and distributed into individual chambers (10 μL each) of a multi-channel microchip preloaded with primers specific for *Mycobacterium tuberculosis* (MTB) and an internal control (IC). The microchip is then incubated at 62 °C for 30 min using a heat block. After incubation, amplified DNA is detected based on precipitate formation, which is visualized using a microscope. (**B**) Representative microscopic images showing the detection of an *M. tuberculosis*-positive sample (1 × 10^5^ CFU/mL) and a negative sample (0 CFU/mL) using the LAMP-MS assay.

The MTB/IC LAMP-MS assay was developed using a multi-channel microchip system preloaded with primers for the simultaneous detection of *Mycobacterium tuberculosis* (MTB) and an internal control (IC). The assay involves adding a mixture containing loop-mediated isothermal amplification (LAMP) reagents and clinical DNA samples directly onto the preloaded chips. The loaded microchips are then incubated at 62 °C for 30 min on a heat block. Following incubation, the presence of amplified DNA is visually confirmed through precipitate formation observed under an optical RV400TMCL microscope (OMAX OMB microscope, Bucheon, Republic of Korea), indicating a positive result (Figure 1).

### 3.2. Limit of Detection of the MTB/IC LAMP-MS Assay

**Figure 2:**
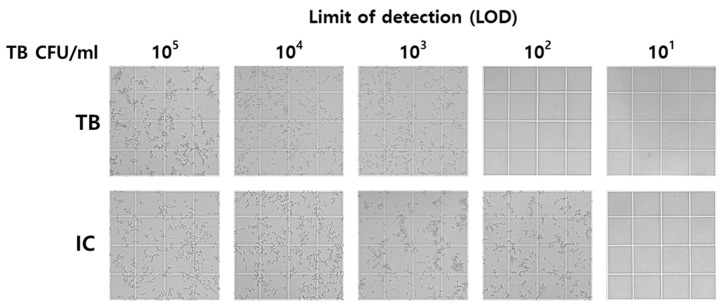
Limit of detection test for the MTB/IC LAMP-MS assay using serial ten-fold dilutions of *M. tuberculosis* DNA samples.

To evaluate the detection sensitivity of the MTB/IC LAMP-MS assay, its limit of detection (LOD) was compared with that of the AdvanSure™ TB/NTM real-time PCR. Table 2 presents the LOD comparison results, while Figure 2 provides a visual representation of the MTB/IC LAMP-MS assay results. The MTB/IC LAMP-MS assay successfully detected *Mycobacterium tuberculosis* at concentrations as low as 1 × 10^3^ CFU/mL, with consistent amplification observed in all twenty replicates. In contrast, the AdvanSure™ TB/NTM real-time PCR demonstrated a detection limit of 1 × 10^2^ CFU/mL, indicating that the MTB/IC LAMP-MS assay had a detection limit one order of magnitude higher than that of the AdvanSure™ TB/NTM real-time PCR. In addition, although signal intensity could be visually observed at different concentrations, the rapid and exponential nature of LAMP amplification leads to saturation, making it difficult to distinguish DNA concentration levels based on visual density. Therefore, the assay is better suited for qualitative rather than quantitative detection.

### 3.3. Comparison of Performance Between the MTB/IC LAMP-MS Assay and the LG AdvanSure™ TB/NTM Real-Time PCR

Clinical sensitivity and specificity were evaluated by comparing the MTB/IC LAMP-MS assay with the LG AdvanSure™ TB/NTM real-time PCR assay using clinical samples (Table 3). Of the 90 total MTB-positive samples tested, 77 were clinical DNA samples, and 13 were sputum samples from which nucleic acids were extracted using two different methods, Qiagen kit (QIAGEN, Hilden, Germany) and Chelex-100/boiling method. The MTB/IC LAMP-MS assay demonstrated an overall clinical sensitivity of 93.3% (84/90) for *Mycobacterium tuberculosis* (MTB), slightly lower than the LG AdvanSure™ TB/NTM real-time PCR assay, which exhibited a sensitivity of 100% (90/90). The sensitivity for the internal control (IC) detection was 95.5% (86/90) for the MTB/IC LAMP-MS assay. Both the MTB/IC LAMP-MS assay and the LG AdvanSure™ TB/NTM real-time PCR showed 100% specificity for 100 non-infected clinical samples. The sensitivity of the internal control (IC) for non-infected clinical samples in both assays was 99% and 100%, respectively. To evaluate whether the LAMP-MS assay could produce reliable results with a rapid and simple extraction method suitable for field use, we additionally analyzed DNA extracted from 13 sputum samples using two different protocols: a standard commercial Qiagen kit and a simplified Chelex-100/boiling method (Table 4). The sensitivity for MTB detection was consistent at 100% for both extraction methods (Qiagen and Chelex-100) across both assays. However, for internal control (IC) detection, the MTB/IC LAMP-MS assay showed a reduced sensitivity of 84.6% (11/13) with the Chelex-100 method, compared to 100% (13/13) when using the Qiagen kit.

### 3.4. Cross-Reactivity Test of the MTB/IC LAMP-MS Assay

The MTB/IC LAMP-MS assay was assessed for cross-reactivity using various respiratory pathogens (viral analytes, bacterial analytes, and NTM species) known to cause clinical symptoms similar to tuberculosis or potentially interfere with diagnostic assays (Table 5). Viral analytes included influenza viruses (A H1, H1N1, H3 and B subtypes), coronavirus (OC43, NL63, and 229E), adenovirus, respiratory syncytial virus (RSV) A, parainfluenza viruses (1–4), and rhinovirus. Bacterial analytes tested were *Legionella pneumophila*, *Streptococcus pneumoniae*, *Klebsiella pneumoniae*, *Pseudomonas aeruginosa*, and *Streptococcus pyogenes*. Additionally, clinically relevant non-tuberculous mycobacteria (NTM) species tested included *M. intracellulare*, *M. avium*, *M. abscessus*, *M. chelonae*, *M. fortuitum*, *M. kansasii*, and *M. massiliense*. The MTB/IC LAMP-MS assay showed no cross-reactivity with any tested pathogens, confirming its high specificity and reliability for MTB detection.

### 3.5. Stability Test of Primer-Preloaded Chips in the MTB/IC LAMP-MS Assay

The stability of the primer-preloaded microchips used in the MTB/IC LAMP-MS assay was evaluated at room temperature over 84 days (Table 6). Throughout this period, chips consistently gave correct results for both MTB-positive samples (positive MTB and IC results) and MTB-negative samples (negative MTB with positive IC results). These findings confirm that primer-preloaded chips remain stable and effective for extended periods without refrigeration, highlighting their practical usability in resource-limited settings.

## 4. Discussion

The MTB/IC LAMP-MS assay developed in this study is a molecular diagnostic method aimed at the rapid on-site detection of tuberculosis (TB). TB remains a global health challenge with high prevalence and mortality rates, necessitating fast and cost-effective diagnostic solutions [24,25,26]. Conventional diagnostic methods, including microscopy, culture, and PCR, have limitations due to high costs, long turnaround times, and the requirement for sophisticated laboratory infrastructure [27,28,29]. The MTB/IC LAMP-MS assay combines microfluidic chip technology with LAMP amplification to enable simple operation and the rapid interpretation of results within 40 min. As shown in Table 7, the total processing time of the MTB/IC LAMP-MS assay is significantly shorter than that of conventional diagnostic methods, especially when combined with the simplified Chelex-100 extraction protocol. This supports its potential as a rapid and accessible alternative for TB diagnosis in point-of-care or resource-limited settings.

Several recent LAMP-based TB diagnostic assays have been developed to enhance accessibility and diagnostic efficiency. Studies have reported LAMP assay sensitivities ranging from 89.5% to 95.2% and specificities between 96.8% and 98.5%, depending on the sample type and nucleic acid extraction method [23,32]. Furthermore, the WHO-endorsed TB-LAMP assay (Eiken Chemical, Japan) is an established molecular diagnostic tool recommended as an alternative to smear microscopy in resource-limited settings. Previous studies have reported its sensitivity between 80% and 95% and specificity between 96% and 99%, depending on the clinical setting [33]. This study presents the MTB/IC LAMP-MS assay, a rapid and cost-effective isothermal amplification-based diagnostic platform for tuberculosis (TB). The assay demonstrated a sensitivity of 93.3% and a specificity of 100% in clinical evaluations, positioning it as a competitive alternative to existing TB diagnostic methods. Furthermore, the MTB/IC LAMP-MS assay utilizes microchips and a microscope, allowing for the visual detection of magnesium pyrophosphate precipitates without the need for expensive fluorescence detection equipment, thereby improving cost efficiency [21,34]. Indeed, the LAMP-MS platform offers a cost-effective alternative to PCR-based diagnostics, requiring minimal instrumentation. PCR necessitates complex laboratory equipment and high operational costs, whereas LAMP-MS only requires a heating block and a microscope for the interpretation of results, leading to significant cost reductions. The Chelex-based DNA extraction method reduces per-sample extraction costs to less than $1 (USD), while the total test cost for LAMP-MS remains between $4 and 6, making it an economically viable option for resource-limited environments [22]. Additionally, the microchip used in this study comes preloaded with primers, allowing for stable storage at room temperature for at least 12 weeks without refrigeration, which further reduces maintenance costs.

As a limitation, the current microchip design includes two separate sample inlets, which could pose a risk of cross-contamination. Future studies should focus on optimizing a single-inlet design to enhance usability and reduce contamination risks. Additionally, the study was conducted with a limited number of clinical samples, requiring larger-scale clinical validation to confirm its robustness and reliability.

In summary, the MTB/IC LAMP-MS assay provides a practical and cost-effective alternative to PCR-based diagnostics, offering rapid, user-friendly operation and high diagnostic accuracy. Its affordability, minimal equipment requirements, and stable storage conditions make it particularly suitable for TB diagnosis in resource-constrained settings. Future studies should focus on optimizing single-inlet microchip designs and conducting large-scale clinical trials across diverse populations to confirm its robustness and field applicability. In addition, although the current assay requires a standard optical microscope for the interpretation of results, we are actively developing a field-deployable diagnostic system that integrates a portable detection module with a simplified nucleic acid extraction function. This would enable the easier implementation of the assay in resource-limited or point-of-care settings.

## 5. Conclusions

The MTB/IC LAMP-MS assay demonstrates high diagnostic accuracy, operational simplicity, and cost-effectiveness, making it a promising alternative for TB detection, particularly in resource-limited settings. Its ability to provide rapid, equipment-minimal diagnoses enhances its field applicability, while its low-cost DNA extraction and stable storage ensure economic feasibility. In clinical evaluations, the assay achieved a sensitivity of 93.3% and a specificity of 100% compared to a commercial real-time PCR kit, demonstrating comparable diagnostic performance to established molecular methods. These results highlight the assay’s potential as a viable alternative for decentralized TB testing. Further large-scale clinical trials and assay optimizations are needed to further validate its practical applicability in TB diagnostics.

## Figures and Tables

**Table 1:** The MTB/IC LAMP-MS primer sets used in this study.

Primer Mix	Target	Name	Sequence (5′-3′)	µM
TB LAMP Primer mix	IS6110 gene	IS6110 F3	GGTCGGAAGCTCCTATGACA	4
		IS6110 B3	TAGGCAGCCTCGAGTTCG	4
		IS6110 FIP	AGGGCTTGCCGGGTTTGATCATGCACTAGCCGAGACGA	32
		IS6110 BIP	CGGTCCATCGAGGATGTCGAGTCGCCGCAGTACTGGTAGA	32
		IS6110 LF	CTCGGTCTTGTATAGGCCGT	10
		IS6110 LB	ACCGCGCGCTGGGTCGA	10
Internal Control (IC) LAMP primer mix	Actin beta gene	IC F3	AGTACCCCATCGAGCACG	4
		IC B3	AGCCTGGATAGCAACGTACA	4
		IC FIP	GAGCCACACGCAGCTCATTGTATCACCAACTGGGACGACA	32
		IC BIP	CTGAACCCCAAGGCCAACCGGCTGGGGTGTTGAAGGTC	32
		IC LF	TGTGGTGCCAGATTTTCTCCA	10
		IC LB	CGAGAAGATGACCCAGATCATGT	10

**Table 2:** Limit of detection test of the LG AdvanSure™ TB/NTM real-time PCR and the MTB/IC LAMP-MS assay for *M. tuberculosis* DNA samples.

TB CFU/mL	LG AdvanSure™ TB/NTM Real-Time PCR		MTB/IC LAMP-MS Assay	
	Ct Values (SD)		P/N	
	MTB	IC	MTB	IC
1 × 10^5^	21.6 ± 0.38	26.4 ± 0.03	P	P
1 × 10^4^	25 ± 0.38	27 ± 0.19	P	P
1 × 10^3^	28.4 ± 0.18	27 ± 0.09	P	P
1 × 10^2^	32.5 ± 0.23	26.6 ± 0.15	N/A	P
1 × 10^1^	N/A	26.7 ± 0.22	N/A	P

Ct = cycle threshold, P = positive results, N = negative results, N/A = not applicable.

**Table 3:** Comparison of clinical performance between TB/IC LAMP-MS and LG AdvanSure™ TB/NTM real-time PCR.

Clinical Samples		LG AdvanSure™ TB/NTM Real-Time PCR Assay		MTB/IC LAMP-MS Assay	
		TB	IC	TB	IC
TB (*n* = 90)	P/N	90/0	90/0	84/6	86/4
	Sensitivity	100%	100%	93.3%	95.5%
	Specificity				
Non-infected (*n* = 100)	P/N	0/100	100/0	0/100	1/99
	Sensitivity		100%		99%
	Specificity	100%		100%	

P = positive results, N = negative results.

**Table 4:** Sensitivity comparison of Qiagen kit and Chelex-100 extraction methods for MTB detection by MTB/IC LAMP-MS and LG AdvanSure™ TB/NTM real-time PCR assays.

Assays		Sensitivity	
		Qiagen Kit	Chelex-100/Boiling
LG AdvanSure™ TB/NTM Real-time PCR Kit	TB	100% (13/13)	100% (13/13)
	IC	100% (13/13)	100% (13/13)
MTB/IC LAMP-MS Assay	TB	100% (13/13)	100% (13/13)
	IC	100% (13/13)	84.6% (11/13)

**Table 5:** Cross-reactivity of the MTB/IC LAMP-MS assay against other human infectious pathogens.

Tested Clinical Samples		MTB/IC LAMP-MS Assay	
		TB	IC
Viral Analytes (*n* = 14)	Influenza A-H1	N	P
	Influenza A subtype H1N1	N	P
	Influenza A-H3	N	P
	Influenza B	N	N
	Coronavirus OC43	N	N
	Coronavirus NL63	N	N
	Coronavirus 229E	N	P
	Adenovirus	N	P
	Respiratory syncytial virus A	N	P
	Parainfluenza virus 1	N	P
	Parainfluenza Virus 2	N	P
	Parainfluenza Virus 3	N	P
	Parainfluenza Virus 4a	N	P
	Rhinovirus	N	N
Bacterial Analytes (*n* = 5)	*Legionella pneumophila*	N	N
	*Streptococcus pneumoniae*	N	N
	*Klebsiella pneumoniae*	N	N
	*Pseudomonas aeruginosa*	N	N
	*Streptococcus pyogenes*	N	N
NTM species (*n* = 7)	*M. intracellulare*	N	N
	*M. avium*	N	N
	*M. abscessus*	N	N
	*M. chelonae*	N	N
	*M. fortuitum*	N	N
	*M. kansasii*	N	N
	*M. massiliense*	N	N

N: N03egative, P: Positive.

**Table 6:** Stability of primer-preloaded chips in the MTB/IC LAMP-MS assay stored at room temperature.

Storage Duration (Days)	MTB/IC LAMP-MS Assay			
	Positive Sample 01	Positive Sample 02	Negative Sample 01	Negative Sample 02
	MTB/IC	MTB/IC	MTB/IC	MTB/IC
1	+/+	+/+	−/+	−/+
3	+/+	+/+	−/+	−/+
7	+/+	+/+	−/+	−/+
14	+/+	+/+	−/+	−/+
28	+/+	+/+	−/+	−/+
56	+/+	+/+	−/+	−/+
84	+/+	+/+	−/+	−/+

**Table 7:** Comparison of total processing time for TB diagnostic methods.

Diagnostic Method	Sample Preparation Time	Amplification & Detection Time	Total Time	Notes
MTB/IC LAMP-MS assay	5~10 min (Chelex-100)	30 min (LAMP) + ~5 min (visual)	~45 min	No thermocycler required; visual detection
Anyplex MTB/NTM real-time assay	~30 min (Qiagen kit)	~120–180 min	~150–210 min	Requires thermocycler and fluorescence detection [30]
Xpert MTB/RIF assay	~15 min (automated cartridge setup)	~90–113 min	~105–130 min	Fully automated, cartridge-dependent system [31]

## Data Availability

The raw data supporting the conclusions of this article will be made available by the authors on request.

## References

[1] Li X., Li Y., Guo L., Chen Y., Wang G., Zhang H. (2025). Tuberculosis Incidence, Deaths and Disability-Adjusted Life Years in Children and Adolescence, 1990–2021: Results from the Global Burden of Disease Study 2021. PLoS ONE.

[2] Lv H., Wang L., Zhang X., Dang C., Liu F., Zhang X., Bai J., You S., Chen H., Zhang W. (2024). Further Analysis of Tuberculosis in Eight High-Burden Countries Based on the Global Burden of Disease Study 2021 Data. Infect. Dis. Poverty.

[3] Sharma S.K., Mohan A., Sharma A. (2012). Challenges in the Diagnosis & Treatment of Miliary Tuberculosis. Indian J. Med. Res..

[4] Ormerod L.P. (2005). Multidrug-Resistant Tuberculosis (MDR-TB): Epidemiology, Prevention and Treatment. Br. Med. Bull..

[5] Mancuso G., Midiri A., De Gaetano S., Ponzo E., Biondo C. (2023). Tackling Drug-Resistant Tuberculosis: New Challenges from the Old Pathogen Mycobacterium Tuberculosis. Microorganisms.

[6] Saderi L., Puci M., Di Lorenzo B., Centis R., D’Ambrosio L., Akkerman O.W., Alffenaar J.-W.C., Caminero J.A., Chakaya J.M., Denholm J.T. (2022). Rapid Diagnosis of XDR and Pre-XDR TB: A Systematic Review of Available Tools. Arch. Bronconeumol..

[7] Zaporojan N., Negrean R.A., Hodișan R., Zaporojan C., Csep A., Zaha D.C. (2024). Evolution of Laboratory Diagnosis of Tuberculosis. Clin. Pract..

[8] Steingart K.R., Henry M., Ng V., Hopewell P.C., Ramsay A., Cunningham J., Urbanczik R., Perkins M., Aziz M.A., Pai M. (2006). Fluorescence versus Conventional Sputum Smear Microscopy for Tuberculosis: A Systematic Review. Lancet Infect. Dis..

[9] Ling D.I., Flores L.L., Riley L.W., Pai M. (2008). Commercial Nucleic-Acid Amplification Tests for Diagnosis of Pulmonary Tuberculosis in Respiratory Specimens: Meta-Analysis and Meta-Regression. PLoS ONE.

[10] Acharya B., Acharya A., Gautam S., Ghimire S.P., Mishra G., Parajuli N., Sapkota B. (2020). Advances in Diagnosis of Tuberculosis: An Update into Molecular Diagnosis of Mycobacterium Tuberculosis. Mol. Biol. Rep..

[11] Chopra K.K., Singh S. (2020). Tuberculosis: Newer Diagnostic Tests: Applications and Limitations. Indian J. Tuberculosis.

[12] Tayal D., Sethi P., Jain P. (2023). Point-of-Care Test for Tuberculosis—A Boon in Diagnosis. Monaldi Arch. Chest Dis..

[13] Shinnick T.M., Starks A.M., Alexander H.L., Castro K.G. (2015). Evaluation of the Cepheid Xpert MTB/RIF Assay. Expert. Rev. Mol. Diagn..

[14] Drobniewski F., Nikolayevskyy V., Balabanova Y., Bang D., Papaventsis D. (2012). Diagnosis of Tuberculosis and Drug Resistance: What Can New Tools Bring Us? [State of the Art Series. New Tools. Number 1 in the Series]. Int. J. Tuberc. Lung Dis..

[15] Ling M.M., Ricks C., Lea P. (2007). Multiplexing Molecular Diagnostics and Immunoassays Using Emerging Microarray Technologies. Expert. Rev. Mol. Diagn..

[16] Becherer L., Borst N., Bakheit M., Frischmann S., Zengerle R., von Stetten F. (2020). Loop-Mediated Isothermal Amplification (LAMP)—Review and Classification of Methods for Sequence-Specific Detection. Anal. Methods.

[17] Soroka M., Wasowicz B., Rymaszewska A. (2021). Loop-Mediated Isothermal Amplification (LAMP): The Better Sibling of PCR?. Cells.

[18] Parida M., Sannarangaiah S., Dash P.K., Rao P.V.L., Morita K. (2008). Loop Mediated Isothermal Amplification (LAMP): A New Generation of Innovative Gene Amplification Technique; Perspectives in Clinical Diagnosis of Infectious Diseases. Rev. Med. Virol..

[19] Nakiyingi L., Nakanwagi P., Briggs J., Agaba T., Mubiru F., Mugenyi M., Ssengooba W., Joloba M.L., Manabe Y.C. (2018). Performance of Loop-Mediated Isothermal Amplification Assay in the Diagnosis of Pulmonary Tuberculosis in a High Prevalence TB/HIV Rural Setting in Uganda. BMC Infect. Dis..

[20] Krishnan S., Syed Z.U.Q. (2022). Colorimetric Visual Sensors for Point-of-Needs Testing. Sens. Actuators Rep..

[21] Choi M., Lee E., Park S., Lim C.-S., Jang W.-S. (2024). Enhanced Point-of-Care SARS-CoV-2 Detection: Integrating RT-LAMP with Microscanning. Biosensors.

[22] Jang W.S., Choi M.K., Choe Y.L., Lim C.S. (2025). Development of a Rapid Malaria LAMP-MS Assay for Diagnosis of Malaria Infections. Sci. Rep..

[23] Kim J., Park B.G., Lim D.H., Jang W.S., Nam J., Mihn D.-C., Lim C.S. (2021). Development and Evaluation of a Multiplex Loop-Mediated Isothermal Amplification (LAMP) Assay for Differentiation of Mycobacterium Tuberculosis and Non-Tuberculosis Mycobacterium in Clinical Samples. PLoS ONE.

[24] Etim N.G., Mirabeau T.Y., Olorode O.A., Nwodo M.U., Izah S.C. (2023). Current Diagnostics Tools of Tuberculosis: Challenges and Opportunities. ES General.

[25] Gupta R., Espinal M.A., Raviglione M.C. (2004). Tuberculosis as a Major Global Health Problem in the 21st Century: A WHO Perspective. Semin. Respir. Crit. Care Med..

[26] Schito M., Peter T.F., Cavanaugh S., Piatek A.S., Young G.J., Alexander H., Coggin W., Domingo G.J., Ellenberger D., Ermantraut E. (2012). Opportunities and Challenges for Cost-Efficient Implementation of New Point-of-Care Diagnostics for HIV and Tuberculosis. J. Infect. Dis..

[27] Petralia S., Conoci S. (2017). PCR Technologies for Point of Care Testing: Progress and Perspectives. ACS Sens..

[28] Basu S., Chakraborty S. (2025). A Comprehensive Review of the Diagnostics for Pediatric Tuberculosis Based on Assay Time, Ease of Operation, and Performance. Microorganisms.

[29] Vaisberg E.A., Lenzi D., Hansen R.L., Keon B.H., Finer J.T. (2006). An Infrastructure for High-Throughput Microscopy: Instrumentation, Informatics, and Integration. Methods Enzymol..

[30] Perry M.D., White P.L., Ruddy M. (2014). Potential for Use of the Seegene Anyplex MTB/NTM Real-Time Detection Assay in a Regional Reference Laboratory. J. Clin. Microbiol..

[31] Kim M.J., Nam Y.S., Cho S.Y., Park T.S., Lee H.J. (2015). Comparison of the Xpert MTB/RIF Assay and Real-Time PCR for the Detection of Mycobacterium Tuberculosis. Ann. Clin. Lab. Sci..

[32] Yan L., Xiao H., Zhang Q. (2016). Systematic Review: Comparison of Xpert MTB/RIF, LAMP and SAT Methods for the Diagnosis of Pulmonary Tuberculosis. Tuberculosis.

[33] Yadav R., Sharma N., Khaneja R., Agarwal P., Kanga A., Behera D., Sethi S. (2017). Evaluation of the TB-LAMP Assay for the Rapid Diagnosis of Pulmonary Tuberculosis in Northern India. Int. J. Tuberc. Lung Dis..

[34] Ghosh K.K., Burns L.D., Cocker E.D., Nimmerjahn A., Ziv Y., El Gamal A., Schnitzer M.J. (2011). Miniaturized Integration of a Fluorescence Microscope. Nat. Methods.

